# Prospect of Human Pluripotent Stem Cell-Derived Neural Crest Stem Cells in Clinical Application

**DOI:** 10.1155/2016/7695836

**Published:** 2016-12-20

**Authors:** Qian Zhu, Qiqi Lu, Rong Gao, Tong Cao

**Affiliations:** ^1^Faculty of Dentistry, National University of Singapore, Singapore 119083; ^2^National University of Singapore Graduate School for Integrative Sciences and Engineering, National University of Singapore, Singapore 117456; ^3^National University of Singapore Tissue Engineering Program (NUSTEP), Life Sciences Institute, National University of Singapore, Singapore 117510

## Abstract

Neural crest stem cells (NCSCs) represent a transient and multipotent cell population that contributes to numerous anatomical structures such as peripheral nervous system, teeth, and cornea. NCSC maldevelopment is related to various human diseases including pigmentation abnormalities, disorders affecting autonomic nervous system, and malformations of teeth, eyes, and hearts. As human pluripotent stem cells including human embryonic stem cells (hESCs) and human induced pluripotent stem cells (hiPSCs) can serve as an unlimited cell source to generate NCSCs, hESC/hiPSC-derived NCSCs can be a valuable tool to study the underlying mechanisms of NCSC-associated diseases, which paves the way for future therapies for these abnormalities. In addition, hESC/hiPSC-derived NCSCs with the capability of differentiating to various cell types are highly promising for clinical organ repair and regeneration. In this review, we first discuss NCSC generation methods from human pluripotent stem cells and differentiation mechanism of NCSCs. Then we focus on the clinical application potential of hESC/hiPSC-derived NCSCs on peripheral nerve injuries, corneal blindness, tooth regeneration, pathological melanogenesis, Hirschsprung disease, and cardiac repair and regeneration.

## 1. Neural Crest Stem Cells

Neural crest stem cells (NCSCs), known as “a fourth germ layer” [1], represent a transient and multipotent cell population that contribute to numerous anatomical structures including peripheral nervous system (PNS), fat tissue, craniofacial skeleton, and cornea [2–4]. NCSCs emerge at the neural plate border between the surface ectoderm and neural ectoderm and migrate extensively to populate diverse derivatives throughout the body [5]. NCSC developmental deficiencies are related to various human diseases including anomalies in facial bone, disorders affecting autonomic nervous system, and malformations of teeth, eyes, and hearts [6]. Previous studies showed that NCSCs were multipotent not only* in vivo* [7–9] but also* in vitro* [10–12]. Because of their multipotent property and developmental significance, NCSCs, which could be isolated from the embryo or generated from human pluripotent stem cells, have great clinical application potential in tissue engineering, drug screening [13–15], and cell therapies for human disease. As it is difficult to obtain NCSCs directly from the embryonic tissue, exploring the alternative sources of NCSCs has become the focus of many scientists.

### 1.1. Strategies for NCSC Differentiation from hESCs/hiPSCs

Lots of studies (listed in Table 1) have shown that NCSCs and their lineages could be derived from human pluripotent stem cells including human embryonic stem cells (hESCs) and human induced pluripotent stem cells (hiPSCs) [16–22]. In 2005, Pomp et al. [16] first reported the generation of NCSCs from hESCs by coculture with mouse stromal PA6 cells, but only a few molecular NCSC markers were tested. In 2007, Lee and his colleagues [17] showed that NCSCs could be derived from hESCs by coculture with stromal cells (MS-5). Identification of NCSCs was confirmed by molecular and clonal analysis, followed by terminal differentiation into peripheral neurons, Schwann cells, and mesenchymal lineages. The* in vivo* transplantation was implemented in both chicken embryos and adult mice to confirm NCSC identity as well. Notably, NCSCs generated in this study possessed self-renewal capacity for several passages, which is similar to the result presented by Jiang's group [18]. In 2009, Hotta et al. [19] developed an efficient method to produce migrating NCSCs from hESCs by using rock inhibitor in the coculture system, and these hESCs-derived NCSCs could differentiate into enteric neurons in the explants of embryonic mouse gut. However, the coculture systems in above studies will result in a mixed population hindering NCSC clinical use. In 2010, one feeder-free system was established by Lee G and his colleagues [20] to generate NCSCs from both hESCs and hiPSCs with defined medium containing Noggin (a BMP inhibitor) and SB431542 (a TGF-*β* inhibitor), which brought a big breakthrough to this area although the population yield was low. In 2011, Menendez et al. [21] achieved a high enrichment of NCSCs from human pluripotent stem cells (hESCs and hiPSCs) in a feeder-free culture system by the inhibition of activin A/nodal pathway combined with Wnt pathway activation. It was demonstrated that the NCSCs generated in this study were capable of forming a great variety of cell types, such as peripheral neurons and mesenchymal stem cells (MSCs). The* in vivo* developmental potential of these NCSCs was confirmed by the transplantation into chicken embryos. This one-step protocol not only is efficient, but also generates NCSCs with stable self-renewal capacity for over 30 passages. Liu et al. [22] established a protocol that could get NCSCs from hESC/hiPSC in 14 days, and Schwann cells with* in vitro* myelination ability that differentiated from NCSCs were first observed in this study. These strategies will facilitate the process of NCSC researches and pave the way for clinical applications of NCSCs.

### 1.2. Differentiation Mechanism of NCSCs

NCSCs are regarded as a valuable cell source for numerous potential cell-based therapies because of their multipotent potential. However, NCSC therapies are currently at the initial stage; understanding the mechanism of generating neural crest-derived lineages from NCSCs is the first but critical step for future clinical use. As the fate decisions of NCSCs are dependent upon signals in the microenvironment [23–25], a given neural crest-derived lineage, such as melanocytes, can be generated from NCSCs with specific factors which either selectively favor NCSC differentiation into a particular lineage or induce NCSCs to a specific cell fate at the expense of other developmental fates [26].

In enteric nervous system (ENS), glial-derived neurotrophic factor (GDNF) is critical for neurogenesis because it promotes the differentiation and survival of NCSCs, while endothelin 3 (ET3) prevents NCSC from differentiating into postmitotic neurons [27]. On the other hand, the development of the melanocytic lineage is favored by stem cell factor (SCF) and ET3 [25, 28]. It has been proved that transforming growth factor beta 1 (TGF*β*1) stimulates smooth muscle differentiation from NCSCs [29]. Consistent with this finding, some* in vivo* studies show that conditional inactivation of TGF*β* signaling in NCSCs could cause cardiovascular defects with the absence of smooth muscle cells, skull defects, and developmental eye disorders [30–32]. What is more, activation of Wnt/*β*-catenin signaling in NCSCs was demonstrated to promote sensory neurogenesis [33], and inactivation of this pathway could also result in loss of the melanocyte lineage [34].

Although lots of other factors still remain to be identified, NCSCs under particular culture conditions can differentiate into the expected neural crest-derived lineages, such as neurons and melanocytes, for therapeutic application.

## 2. Clinical Applications of NCSCs

**Figure 1:**
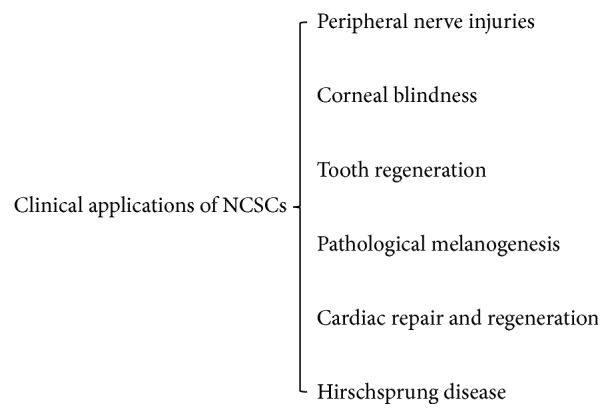
A summary of NCSC clinical applications.

Great success has been achieved in the field of cell-based therapies and organ reconstructions with utilization of adult stem cells including MSCs [35] and in particular adipose-derived stem cells [36]. MSCs as the typical stem cells for clinical research are present in many organs and tissues including bone marrow, skin, fat, and umbilical cord, and they can differentiate into adipocytes, osteoblasts, and chondrocytes [35, 37]. In comparison with MSCs, the quantity of NCSCs that can be isolated from adults is relatively rare [38], but they possess a much broader developmental potential including differentiation into MSCs, which is only topped by ESCs [26]. The discovery of easily accessible sources for NCSC isolation from adult tissues, such as skin and dental pup [38], is of great importance for NCSC clinical application, as it meets the requirement for low invasive isolation procedures. For example, Pisciotta et al. [39] demonstrated that one subpopulation of human dental pulp stem cells possessed characteristics similar to NCSCs including multilineage differentiation potential. These NCSC sources are available throughout adulthood, but NCSCs isolated from adults are still rare and display more restricted multipotency and self-renewal capacity compared to their embryonic counterparts [38]. To tackle such problems, hESCs/hiPSCs are often used as an unlimited cell source to generate NCSCs for their clinical use since it is not feasible to isolate endogenous embryonic population directly. MSCs as the regenerative cells have already been used in lots of clinical trials [40], while the potential clinical applications of NCSCs from human pluripotent cells are still in its infancy now. In spite of some ethical or safety concerns, hESC/hiPSC-derived NCSCs will have wider clinical applications than other stem cell populations including MSCs with further researches because of their astonishing multipotentiality. In addition, hiPSC-derived NCSCs open a door to overcome potential histocompatibility issues during the transplantation. Here, some potential clinical applications of hESC/hiPSC-derived NCSCs are listed in Figure 1 including regeneration and reconstruction of different tissues and new treatments for NCSC-related diseases.

### 2.1. Peripheral Nerve Injuries

Damage to peripheral nerves primarily caused by traumatic injury or surgical manipulation is very common. Peripheral nerve injuries ranging from mild (some nerve deficits) to severe (loss of major function) can significantly compromise the quality of patients' life [41]. It was reported that more than 50,000 repair procedures for peripheral nerves were performed in 1995. However, the actual number of peripheral nerve injuries probably exceeded this figure, as not all peripheral nerve damage could be repaired [42]. Despite novel surgical techniques and modern treatment, functional and structural restoration of peripheral nerves remains a great challenge. Therefore, there is a need to improve the clinical outcomes of peripheral nerve repair. Stem cell-based therapies with tissue-engineered scaffolds are now being developed to accelerate the regeneration of peripheral nerve injuries [43, 44].

It is known that, for peripheral nerve repair, one functional engineered construct involves four central components including support cells, a scaffold supporting axonal migration, the extracellular matrix, and growth factors [42]. Although the underlying mechanisms for regeneration of the injured peripheral nerves remain unclear, NCSCs can be used as a promising stem cell type for peripheral nerve repair. This finding is supported by a study of Vasyliev et al. [44], which reported that after implantation of neural crest-derived multipotent stem cells into the transected region of mice, the intensity of nerve regeneration in this injured region was increased compared with that in mice without transplantation. This work indicated that NCSCs possess a pronounced biological property to promote nerve repair by differentiating into Schwann cells that are indispensable in peripheral nerve regeneration, as Schwann cells not only provide a support for axon migration but also secrete neurotrophic factors to promote nerve growth [42, 45]. Severe skin injuries always come with damage to peripheral nerves, so innervated 3D skin reconstructions could be developed as optimal skin substitutes for the patients to restore neurological function. Sensory neurons in skin models could not only potentially restore the sensibility of the skin [46], but also accelerate keratinocyte reepithelialization through secreting the neuropeptide substance P [47]. As mature neurons are difficult to obtain from human bodies and do not proliferate significantly both* in vitro* and* in vivo*, hESC/hiPSC-derived NCSCs, which can provide a large number of peripheral neurons for the innervated dermal substitutes, have great clinical significance for human skin transplantation.

Since NCSCs can differentiate into Schwann cells and neurons for peripheral nerve regeneration, cellular therapies based on hESC/hiPSC-derived NCSCs will be attractive candidates to repair peripheral nerve injuries.

### 2.2. Corneal Blindness

The cornea is a transparent organ which not only protects the eyeball from insults, but also transmits visible light to produce images on the retina. The cornea is comprised of five main layers, including the outermost corneal epithelium, Bowman's layer, the keratocyte-populated corneal stroma, Descemet's membrane, and the inner corneal endothelium [48]. The corneal stroma that constitutes around 90% of the cornea's thickness is formed by a transparent extracellular matrix, which is uniquely secreted by keratocytes [49]. Surgical or accidental injuries to corneal stroma will lead to corneal scarring or even corneal blindness because of fibrotic deposit in stroma. In addition, the corneal endothelium, which consists of hexagonal corneal endothelial cells (CECs), is physiologically the most significant barrier in cornea, as it plays an obligatory role in regulating corneal hydration and maintaining corneal transparency and thickness [50, 51]. Corneal endothelial dysfunction caused by infection or trauma will disturb the pumping function of corneal endothelium and further cause corneal edema and even visual blindness [51].

Millions of people in the world are affected by corneal blindness, and currently the surgical cornea transplantation is the only effective approach to restore corneal function and clarity. But this therapy is still with well-known limitations, such as surgical complications, graft failure, and shortage of qualified donor cornea [52]. Since the donor corneal tissues are in a short supply, it is critical to develop alternative approaches to restore vision instead of keratoplasty. Although tissue-engineered corneal equivalents have been explored, there are serious drawbacks in their application such as strong inflammatory responses and unsatisfactory tissue regeneration [53]. Recently, stem cell therapy as another alternative technique to restore corneal transparency and function has attracted more and more attention [4, 53–55].

It is known that both corneal keratocytes and CECs are derived from NCSCs [3, 4, 56]. Therefore, NCSC-based cell therapy would ideally be used to produce autologous corneal equivalents without immune rejection. Hertsenberg and Funderburgh [57] used a two-step method to generate keratocytes from hESCs by initial differentiation into NCSCs and subsequent induction toward corneal keratocytes. In addition, Ju et al. [4] proved the feasibility of differentiating rodent NCSCs into functional CECs, and the effect of CECs was confirmed by transplanting them to the rat model with corneal endothelium deficiency. Histological examination showed that these polygonal CECs formed a monolayer to cover the Descemet's membrane in rats, which is consistent with results of confocal microscopy. In 2015, Chen et al. [58] induced mouse ESCs and iPSCs into CECs by directing mouse pluripotent stem cells into NCSCs at first and subsequently toward CECs, which were characterized by morphology, immunocytochemistry (ICC), and quantitative PCR (QPCR). Moreover, McCabe and his colleagues [59] developed one protocol to generate CECs from hESCs via NCSCs by defined factors. Microarray analysis revealed that the CECs from hESC-derived NCSCs showed close similarity to primary adult human CECs.

In conclusion, it is now possible to generate corneal keratocytes or CECs from hESC/hiPSC-derived NCSCs in a two-step process* in vitro*, which provides promising opportunities to develop suitable corneal alternatives for corneal blindness treatment instead of donor cornea.

### 2.3. Tooth Regeneration

Teeth, the hardest tissues in the human body, are comprised of enamel, dentin, pulp, cementum, and periodontal ligament. Teeth have diverse functions including chewing, pronunciation, and aesthetics, so tooth loss has negatively physical and emotional impacts on patients, which deteriorates their psychosocial well-being and self-esteem [60–62]. Currently, many seniors are at risk for tooth loss with advancing age. The prevalence of congenitally missing teeth (CMT) may also increase because of the changes in evolution and the improvement in diagnostic tools and criteria [63, 64].

The current treatments for tooth loss or absence typically involve dental implants or dentures made from synthetic biomaterials. However, there are many complications associated with denture therapy including denture-related oral ulcers and stomatitis [65]. The application of prosthetic implants may also result in implant failures and complications, which are mainly attributable to bacterial infection, impaired healing, and overload [66]. To overcome these issues, candidate approaches have been explored. Considerable progress has been made currently in the field of bioengineered rodent tooth replacement [67–70] that suggests the great potential about human tooth regeneration. One of the main barriers in this field is to search for a suitable and stable cell source for regenerating human teeth.

Mammalian teeth development is controlled by two types of cells: ectodermal epithelial cells, which give rise to ameloblasts, and NCSCs, which contribute to most of the dental tissues including dentin matrix, dental pulp, cementum, and periodontal ligament [71–73]. In 2008, Xu's lab [74] indicated that cranial neural crest cells were capable of* in vitro* odontogenesis with adult extracellular matrix. These cranial neural crest-derived odontoblast-like cells were characterized by morphology, alkaline phosphatase activity, expression of odontoblast markers, and formation of mineralized nodules. Recently, Seki et al. [75] reported one method to induce mouse iPSC-derived neural crest like cells (iNCLCs) into odontoblast-like cells by gene transfection of bone morphogenetic protein 4 (Bmp4) and Pax9. In addition, it was notable that there was no teratoma formation when iNCLCs and transfected iNCLCs were injected subcutaneously in mice. These results suggest that NCSCs derived from pluripotent stem cells could be a safe and unlimited cell source to generate odontoblasts for tooth regeneration. Moreover, it was shown that cementoblast-like cells were generated from cranial neural crest cells* in vitro* when treated with dental follicle cell conditioned media [76]. Due to the vital role that NCSCs play in tooth development, more and more efforts will be invested into the differentiation of dental tissue-forming cells from hESC/hiPSC-derived NCSCs in the future.

In summary, hESC/hiPSC-derived NCSCs in combination with ectodermal epithelial cells are considered as an optimal and promising cell source for the whole tooth regeneration. Thus, the regeneration of human teeth would become feasible in the near future by using hESC/hiPSC-derived NCSCs.

### 2.4. Pathological Melanogenesis

In vertebrates, melanocytes derived from NCSCs are melanin-producing cells. Human melanocytes, which are primarily located in the hair follicles and skin, not only are essential for skin and hair pigmentation, but also protect the skin from ultraviolet irradiation [77]. It is notoriously known that the abnormal melanocytes are related to a wide range of severe diseases including vitiligo, albinism, and melanoma [78, 79]. Vitiligo characterized by the depigmentation of the skin and hair is a worldwide common pigmentary disorder and may significantly affect the patients' self-esteem and life satisfaction [80]. As one of the deadliest aggressive skin cancers known for its high metastasis ability, melanoma is getting more prevalent over the past several decades [79, 81–83]. To address these issues, it is crucial to understand melanocyte development and function. However, the detailed function and developmental mechanisms of human melanocytes are still absent at present because of the difficulties in obtaining sufficient melanocytes for research models. In addition, the shortage of melanocytes also limits their clinical applications for melanocyte-related diseases including cell transplantation and drug screening. Therefore, it is important to explore efficient ways to generate melanocytes that could be not only used for laboratory researches, but also used as a cell source for clinical therapeutics.

Several groups are now actively investigating the melanocyte differentiation techniques that may provide a stable cell source for melanocyte researches. Shakhova and Sommer [84] have established a differentiation approach to generate melanocytes from embryonic NCSCs in 10 days by the addition of a cocktail of specific growth factors such as ET-3 and mouse SCF. Their study showed that after 10-day differentiation, the majority of NCSCs presented a melanocytic phenotype indicating the* in vitro* differentiation ability of NCSCs into melanocytes. In 2011, Kawakami lab [85] developed a method to induce hiPSCs into human melanocytes* in vitro* through an intermediate phase of NCSCs. Several weeks after differentiation, established melanocyte markers were positive in these hiPSC-derived-melanocytes, and melanosome formation could be detected as well. The effectiveness of this* in vitro* differentiation system was further confirmed by DNA microarray, which showed a high-level similarity of global gene expression between these pigment cells and normal human foreskin-derived epidermal melanocyte. Nissan et al. [86] also supported the* in vitro* availability of human pluripotent stem cells committed to the melanocytes via neural crest stage. Moreover, human melanocytes derived in this study exhibit both phenotypical and functional characteristics of their adult counterparts. Recently, Mica and colleagues [87] established a stepwise differentiation strategy to obtain functional melanocytes from human pluripotent stem cells by initial NCSC induction, followed by specification of melanoblasts and eventual differentiation into mature pigmented cells. Besides the analysis of gene and protein expression, electron microscopy revealed that lots of pigmented melanosomes were present in the melanocytes generated with this protocol. Intriguingly, an organotypic skin model was established to characterize the function of these melanocytes, which were found to be home to the correct location in the basement membrane zone. The above protocols for melanocyte generation allow the identification of factors regulating the melanocyte development and function. The possible genetic and cellular mechanisms involved in pathological melanogenesis could also be characterized by comparing the melanocytes generated from these protocols with the melanocytes from normal population and patients with pigment cell disorders. Based on this research model, it is possible to predict the incidence or prevalence of melanocyte-related diseases in population in the future.

Access to an unlimited melanocytes resource may also contribute to cell-based therapies for hypopigmentation disorders and therapeutic drug screening for melanocyte relevant diseases. Melanocyte transplantation has been experimentally used as an adjunct cell therapy for vitiligo for years [88]. As early as 1987, Lerner et al. [89] reported the implantation of autologous cultured human melanocytes. They first isolated melanocytes from the normally pigmented skin of the patient with piebaldism and then expanded these normal melanocytes in culture, followed by transplanting them into the hypopigmented skin area of this patient. Six month later, excellent repigmentation was noticed in the grafted area. Melanocytes generated from hESC/hiPSC-derived NCSCs thus can be used as novel approaches to provide enough grafting material for the patients with hypopigmentation disorders. In addition, the unlimited cell source of melanocytes represents a potential tool for drug testing in melanocyte-related diseases. Mica et al. [87] offered an insight into genetic pigmentation defect models and demonstrated that melanocytes derived from special iPSC of patients with Hermansky-Pudlak syndrome and Chediak-Higashi syndrome were capable of reflecting the biological nature of these pigmentation disorders. Such disease-specific melanocytes can be particularly used to identify potential drugs that can reverse pigmentation disorders and correct relevant function. Therefore, it sets a stage for exploring candidate drugs for potential therapeutic intervention.

Taken together, hESC/hiPSC-derived NCSCs have been proved as a useful tool to produce a large number of human melanocytes for basic research, clinical treatment, and drug discovery. Recent advances in the differentiation of melanocytes from hESC/hiPSC-derived NCSCs have offered exciting opportunities to investigate the developmental mechanisms of human melanocytes with specific phenotypes in normal and pathological conditions. These finding can now provide novel platforms for a broad range of clinical applications, including pathological melanogenesis modeling, potential cell therapies, and therapeutic drug testing. Finally, the clinical application of melanocytes generated from hESC/hiPSC-derived NCSCs will benefit millions of patients suffering from various melanocyte-associated disorders.

### 2.5. Cardiac Repair and Regeneration

As the first functional organ developed in mammalian, the heart provides sufficient oxygen and nutrients for embryogenesis. Although the heart originates from mesoderm, NCSCs are crucial for the heart development [90]. As a subpopulation of cranial neural crest cells, cardiac neural crest cells (CNCCs) participate in cardiac valve formation and cardiac parasympathetic innervation. The septation of the outflow tract in heart also critically depends on the development of CNCCs [91, 92].

Notably, defects of CNCCs will lead to various congenital heart diseases such as tetralogy of Fallot, CHARGE syndrome, Noonan syndrome, and double outlet right ventricle [93, 94]. Whereas these heart malformations are rare, they are always life threatening for patients. Therefore, it is imperative to explore potential therapies for these specific defects. NCSCs derived from hESC/hiPSC can serve as a model to investigate the dynamics of relevant congenital heart diseases and subsequently find ways to prevent or correct them.

Furthermore, hESC/hiPSC-derived NCSCs are capable of providing a pool of important cardiac progenitor cells for human heart repair and regeneration. In mammalian, CNCCs reside in the heart as dormant multipotent stem cells after migration, which can give rise to various cell types involved in cardiogenesis including aortic smooth muscle cells, glia cells, and neurons [95]. In 2008, El-Helou et al. [96] demonstrated that the nestin-expressing cells isolated from infarcted hearts were derived from neural crest. To investigate the biological function of theses nestin-positive cells in the infarct regions, this subpopulation was fluorescently labelled and subsequently injected into the hearts of rats with myocardial infarction. This* in vivo* transplantation model showed that the injected cells were detected exclusively in the injured area and contributed to the cardiovascular structure formation. Recently, a study by Tamura et al. [97] revealed that the adrenergic cells from NCSCs were increased after heterotopic cardiac transplantation in mice, which was tightly coupled to the activation of intrinsic cardiac adrenergic function in transplanted murine hearts. NCSCs thus are essential for neonatal and adult cardiac regeneration in human. Stem cell therapies based on hESC/hiPSC-derived NCSCs offer an exciting prospect for clinical heart repair after diseases or injuries.

Differentiation protocols that drive hESC/hiPSC-derived NCSCs to particular cardiac progenitors provide not only a valuable tool to promote the healing process following heart injuries, but also a potential cell source participating in human heart regeneration. What is more, hESC/hiPSC-derived NCSCs can be used as a suitable model for uncovering the underlying mechanisms of NCSC-associated congenital heart diseases as well as developing clinical treatments for these cardiac malformations.

### 2.6. Hirschsprung Disease

Hirschsprung disease (HSCR) that occurs in approximately 1 in 5000 newborns is a common intestinal mobility disorder characterized by the absence of enteric ganglion cells in distal regions of the colon. As a life-threatening disease of pediatrics, HSCR can lead to tonic contraction of aganglionic bowel, intestinal blockage, and even fatal dilation of the colon (megacolon) [98]. Surgical operation to remove the aganglionic segment of the colon is currently used for children with HSCR. However, gastrointestinal dysfunction of the remaining digestive tract persists in the patients subjected to this life-saving treatment [99]. Furthermore, patients suffering from total colonic aganglionosis (TCA) usually undergo lots of complications, and reoperation is often required [100]. Due to the limited therapeutic strategies for HSCR at present, it is necessary to develop novel and efficient drug-based and cell-based therapies for the patients with HSCR.

HSCR is caused by defects in the development of ENS that is derived from vagal and sacral neural crest [15, 101]. Given the ability of NCSCs to form ENS, NCSCs could not only be a useful tool to study mechanisms underlying HSCR, but also provide feasible alternative treatments for HSCR. In 2003, Iwashita and his colleagues [102] found that compared to whole-fetus RNA, genes implicated in HSCR showed higher expression levels in gut NCSCs, and mutations in these HSCR genes could result in severe ENS defects by affecting gut NCSC function. This result indicated that HSCR is associated with defects in the function of NCSCs. NCSCs generated from hESC/hiPSC can serve as a stable cell source to study the cellular and molecular mechanisms of HSCR. In addition, hESC/hiPSC-derived NCSCs as stem cell transplants are capable of repopulating the ENS for HSCR. In 2009, Hotta et al. [19] showed that hESCs-derived neural crest like cells could differentiate into enteric neurons after transplanting them into mouse gut tissue. A recent report illustrated that enteric neural crest (ENC) precursors derived from hESCs possess the ability to repopulate the colon in the host and rescue the mortality of HSCR mice [15]. What is more, mutant ENC precursors from human pluripotent stem cells can be utilized as a platform to perform candidate drug screening. Since null mutations in the genes encoding endothelin-3-endothelin receptor B (EDNRB) are responsible for a subset of HSCR patients [103], Fattahi et al. [15] used hESCs-derived EDNRB^−/−^ ENC precursors as a HSCR model to screen and identify compounds that could rescue the migration defects associated with HSCR.

Many challenges need to be overcome before applying hESC/hiPSC-derived-NCSCs into HSCR clinical trials; nonetheless, cell-based and drug-based therapies for HSCR by using hESC/hiPSC-derived-NCSCs remain a promising prospect.

## 3. Conclusions and Discussion

NCSCs are a multipotent cell population with the capacity to differentiate into a diverse array of cell types. There are many congenital diseases associated with maldevelopment of NCSCs, some of which are even life-threatening. As human pluripotent stem cells can serve as an unlimited and stable cell source to generate NCSCs, hESC/hiPSC-derived NCSCs will be a powerful tool to study the underlying causes of relevant diseases and explore new therapies to prevent or correct them. In addition, hESC/hiPSC-derived NCSCs provide a pool of various cells, which are large enough for a wide range of clinical organ repair and regeneration.

For future NCSC clinical research, the safety issue of NCSCs seems to be a chief problem to consider due to the versatile but dangerous nature of NCSCs. The study of Seki et al. [75] showed that no teratoma was formed after iNCLC transplantation into mice suggesting the safety of NCSCs derived from pluripotent stem cells. This is in line with the previous report of Wang et al. [104], which indicated that hiPSC-derived NCSCs could be directly utilized for tissue engineering because no teratoma formation was discovered in rats following the transplantation of NCSCs for up to one year. However, the possibilities for hESC/hiPSC-derived NCSC deviant differentiation into unwanted neural crest-derived lineages after* in vivo* transplantation must be evaluated thoroughly before their clinical applications. The* in vivo* differentiation fate of NCSCs largely depends on the microenvironment encountered, and there are diverse tumors of neural crest origin including malignant melanoma and neurofibromatosis [105]. It was found that the maintenance or activation of NCSC genetic program was an important pathogenic feature of Ewing sarcoma family tumors (ESFT), which are a group of aggressive tumors occurring in bones and soft tissues, because the results of gene expression profiling showed ESFT were genetically closely associated with NCSCs [106]. Since the application of NCSCs might result in various neural rest-derived cancers, such as neuroblastoma [107, 108] and clear cell sarcoma [109], potential risk of NCSCs should be seriously taken into account before NCSC clinical therapy. Although obstacles such as safety and technical complexities have to be addressed before extensive clinical application, hESC/hiPSC-derived NCSC-based therapies are highly promising. Due to the importance of NCSCs, more efforts will be invested in this field to benefit all humanity.

## Figures and Tables

**Table 1:** A summary of studies about NCSC differentiation from human pluripotent stem cells.

Reference	Cell source	Culture system	Supplemental factors	The yield of NCSCs	Time length	Cells induced from NCSCs *in vitro*	*In vivo* experiment	Remarks
Pomp et al., 2005 [16]	hESCs	Coculture (PA6)	Serum	42.3% ± 5.5%	1 week	Peripheral sensory and sympathetic neurons	Null	First paper about NCSC induction from hESCs, but only a few molecular markers were tested

Lee et al., 2007 [17]	hESCs	Coculture (MS-5)	Culture were switched to N2 media with serum after neural rosettes emerge from the differentiating hESCs	~30%	~1 month	Peripheral nerve system and mesenchymal stem cells	Chicken embryos and adult mice	First report using *in vivo *transplantation to confirm the identity of hESC-derived NCSCs, which were capable of self-renewal for several passages

Jiang et al., 2009 [18]	hESCs	Coculture (PA6)	Serum	55.3% ± 10.6% of the hESC colonies expressing P75	1 week	Peripheral neurons, glial cells, and myofibroblasts	Chicken embryos	P75^+^ cells after sorting showed self-renewal capacity

Hotta et al., 2009 [19]	hESCs	Coculture (MEF)	Noggin, Y27632	97.7% ± 0.7% of the migrating cells were P75^+^	~20 days	Enteric neurons	Quail embryos	First study reporting the use of Y27632 to induce hESCs into migrating NCSCs

Lee et al., 2010 [20]	hESCs hiPSCs	(1) Coculture (MS-5) (2) 2D monolayer (Matrigel)	Noggin, SB431542 in defined media	(1) ~30% (2) 15%~40%	(1) ~30 days (2) 12 days	Peripheral neurons, Schwann cells, and myofibroblasts	Null	A feeder-free system was developed to generated NCSCs from both hESCs and hiPSCs

Menendez et al., 2011 [21]	hESCs hiPSCs	2D monolayer (Geltrex)	SB431542, BIO	80%~99%	~2 weeks	Peripheral neurons and mesenchymal stem cells	Chicken embryos	This one-step protocol not only is efficient, but also generates NCSCs with stable self-renewal capacity for over 30 passages

Liu et al., 2012 [22]	hESCs hiPSCs	2D monolayer (Geltrex)	PA6 conditioned media containing FGF2, Rock inhibitor, and ascorbic acid	~46%	14 days	Peripheral neurons, Schwann cells, and mesenchymal lineages	Chicken embryos	First report showed *in vitro *myelination ability of Schwann cells differentiated from hESC/hiPSC-derived NCSCs

MEF: mouse embryonic fibroblasts; FGF2: fibroblast growth factor 2.
